# EP18 Testicular sarcoidosis: a challenging diagnosis

**DOI:** 10.1093/rap/rkaa052.017

**Published:** 2020-11-03

**Authors:** Vishit Patel, Sanketh Rampes, Deepak Nagra, Benjamin Clarke, James Galloway

**Affiliations:** r1 Faculty of Life Sciences & Medicine, King’s College London, London, United Kingdom; r2 Centre for Rheumatic Diseases, King’s College London, London, United Kingdom

## Abstract

**Case report - Introduction:**

Sarcoidosis is an inflammatory systemic disorder that is characterised by the formation of immune granulomas. Lung involvement is seen in about 90% of patients but extrapulmonary sarcoidosis can be a clinically challenging manifestation. Despite the majority remitting in three years, a considerable proportion (10-30%) develop chronic disease requiring continuous treatment. The development of extrapulmonary disease can be prior to, after or concomitant with pulmonary disease. Although cardiac, ophthalmic, neurological, and musculoskeletal manifestations have been described elsewhere, testicular involvement remains a rare phenotype of the disease which is poorly understood.

**Case report - Case description:**

A 36-year old male patient born in Jamaica, presented in 2017 with unilateral left-sided testicular pain and enlargement measuring 20 x 16 x 18mm on ultrasound. This was initially suspected of malignancy or an atypical infection. A subsequent CT chest, abdomen and pelvis demonstrated nodal evidence of hilar, internal mammary and mediastinum involvement. Blood tests were unremarkable other than a raised lactate dehydrogenase (248 IU. mL (NR < 240)), low testosterone (5.2nmol/L (NR 10-30)) and androgen index (11.6 (NR 25-90).

The patient underwent an orchidectomy and prosthesis, histological sampling demonstrated idiopathic granulomatous orchitis with features consistent of sarcoidosis. Malignancy could be excluded, and the absence of characteristic histochemical staining patterns favoured sarcoid to other differential diagnoses. He was efficaciously treated after a year’s interval with prednisolone, which was gradually weaned from 40mg.

However, eighteen months later, the patient returned complaining of right testicular pain. Ultrasound showed inflammation and enlargement of the testicle. PET-CT supported a recurrence of testicular involvement along with systemic disease involvement including neuropathy. His serum angiotensin-converting enzyme was raised at 118 IU/L (NR8-52) and responded similarly to high dose corticosteroid treatment. Additionally, an MRI brain showed neurosarcoid in the superior sagittal sinus tracking along the right transverse sinus.

Currently the patient is maintained on subcutaneous methotrexate 25mg disease modifying anti-rheumatic drug (DMARD) monotherapy. He has diabetes attributed to prolonged corticosteroid use over the last three years. As a result of his testicular involvement, the patient suffers from erectile dysfunction and hypogonadism with low testosterone production. Chronic pain and neuro-sagittal involvement have contributed to difficulties in achieving treatment targets with increased malaise, fatigue and poor cognition all compounding his disease further. This has posed a challenge as to whether this is due to the disease or the patients use of cannabis or a combination of the two.

**Case report - Discussion:**

Testicular sarcoid is a rare presentation in which only around 0.2% of all sarcoidosis cases are diagnosed. The epididymis, vas deferens and testis can all be involved. Presentation can mimic that of infections such as tuberculosis mycobacterium and malignancy with uni- or bilateral testicular involvement. Challenges arise, as in this case, when the patient presents with genito-urinary involvement in the absence of more systemic features which may favour extra-pulmonary manifestations.

The diagnosis and treatment can be difficult, as corticosteroid response tends to be most effective at high doses and disease modifying anti-rheumatic drugs (DMARDs) such as methotrexate have evidence for pulmonary and extra-pulmonary sites.

Our patient developed more systemic features at a later stage. The diagnosis was challenging and there was an interval of a year between the orchidectomy and commencement of steroids. This shows the ongoing difficulties in diagnosing patients with sarcoidosis and the delays that occur in treatment initiation. Testicular involvement has created debilitating symptoms for the patient with chronic pain affecting his ability to sit and ride his bike for extended periods. Involvement of the other testicle, ongoing low testosterone production and erectile dysfunction have posed difficult discussions around future fertility. It is encouraging that he has managed to wean from corticosteroid and methotrexate seems to be controlling the disease quite well. The evidence relating to best possible DMARD treatment in testicular sarcoid is still scarce and is an interesting point of discussion. There have been co-existent reports of testicular tumours in sarcoid, and as most of these patients are seen by surgical specialties, further work must heighten awareness of sarcoid in this cohort of patients.

**Case report - Key learning points:**

We feel this is an unusual presentation of sarcoidosis; a patient who presented with primary testicular involvement and is pertinent to the topic of extrapulmonary disease manifestation. Despite appropriate treatment, he developed recurrence within the other testis two years later.

Testicular involvement as a manifestation of extra-pulmonary sarcoidosis is rare and difficult to treat. There are limitations in treatment beyond corticosteroid, although DMARD therapy with methotrexate being favoured in case reports listed elsewhere. It is difficult to differentiate from malignancy and infection, and as a result often requires complete removal of the testis for histological interpretation.

This can lead to hypogonadism and may affect long-term fertility which has significant psychosocial impacts for patients. There is an association between sarcoidosis and the development of co-morbidities which carries significant long-term complications. The relationship between long-term corticosteroid use and ethnicity in the development of co-morbidities in patients with sarcoidosis is poorly understood.

Medical and surgical specialties that manage most cases involving testicular masses may not be aware of the differentials such as sarcoid; clinicians may therefore be exposed to a degree of cognitive bias in these cases. We propose this as an example to improve framing of similar cases for clinicians in the future. From a patient’s perspective, similar cases require careful discussion to explore potential management options and their outcomes as the impact may be life changing.

